# Impact of High Salt Independent of Blood Pressure on PRMT/ADMA/DDAH Pathway in the Aorta of Dahl Salt-Sensitive Rats

**DOI:** 10.3390/ijms14048062

**Published:** 2013-04-12

**Authors:** Yu Cao, Jian-Jun Mu, Yuan Fang, Zu-Yi Yuan, Fu-Qiang Liu

**Affiliations:** Department of Cardiovascular Medicine, First Affiliated Hospital of Medical College of Xi’an Jiaotong University, No. 277 Yanta West Road, Xi’an, Shaanxi 710061, China; E-Mails: caoyu1379934@163.com (Y.C.); zuyiyuan@mail.xjtu.edu.cn (Z.-Y.Y.); Liufuqiang0909@163.com (F.-Q.L.)

**Keywords:** endothelial dysfunction, asymmetric dimethylarginine, dimethylarginine, dimethylaminohydrolase, endothelial nitrite oxide synthase, oxidative stress

## Abstract

Endothelial dysfunction participates in the development and progression of salt-sensitive hypertension. Asymmetric dimethylarginine (ADMA) is an endogenous inhibitor of nitric oxide synthase (NOS). The objectives of this study were to investigate the impact of a high salt diet on the PRMT/ADMA/DDAH (protein arginine methyltransferases; dimethylarginine dimethylaminohydrolase) pathway in Dahl salt-sensitive (DS) rats and SS-13^BN^ consomic (DR) rats, and to explore the mechanisms that regulate ADMA metabolism independent of blood pressure reduction. Plasma levels of nitric oxide (NO) in DS rats given a high salt diet and subjected to intragastric administration of hydralazine (SH + HYD group) were lower than those given a normal salt diet (SN group). There were significant decreases in expression and activity of dimethylarginine dimethylaminohydrolase (DDAH) and endothelial NO synthase (eNOS) in DS rats given a high diet (SH group) in comparison to the SN group. The activity of DDAH and expression of eNOS in the SH + HYD group decreased more significantly than SN group. The mRNA expression of DDAH-1 and DDAH-2 were lowest in the SH group. The results suggest that salt, independent of blood pressure, can affect the PRMT-1/ADMA/DDAH system to a certain degree and lead to endothelial dysfunction in Dahl salt-sensitive rats.

## 1. Introduction

Endothelial dysfunction is involved in the pathogenesis of cardiovascular diseases and participates in the development and progression of salt-sensitive hypertension [1]. Nitric oxide (NO) plays an important role in the regulation of vasodilatation [2,3]. A decline in NO bioavailability can contribute to endothelial dysfunction. One important molecular mechanism is an endogenous competitive inhibitor of nitric oxide synthase (NOS), *N^G^*,*N^G^*-dimethylarginine (asymmetrical dimethylarginine, ADMA) [4]. A growing body of evidence suggests that ADMA is an independent risk factor in the pathophysiology of salt-sensitive hypertension [5–7].

ADMA is synthesized endogenously during the methylation of protein arginine residues by protein arginine methyltransferases (PRMTs). There are two broad types of PRMTs: PRMT-1 catalyze the formation of ADMA, whereas PRMT-2 methylate both of the guanidino nitrogens and thus result in the formation of symmetric dimethylarginine (SDMA); SDMA has no inhibitory activity [8,9]. In the cardiovascular system, ADMA is generated by PRMT-1, which is expressed in the heart, smooth muscle cells, and endothelial cells. Almost 80%–90% of endogenous ADMA is metabolized by DDAH, of which there are two isoforms: DDAH-1 and DDAH-2. DDAH1 is most highly expressed in the brain and kidney. DDAH-2 is expressed strongly in the endothelium and vascular smooth muscle cells, found mainly in tissues containing endothelial NOS (eNOS) [10]. In cardiovascular tissues, both DDAH isoforms are expressed in the same cell [11,12]. There are still some disputes about the relative contribution of each DDAH isoform to total methylarginine metabolism.

Most animal experiments and population investigations have shown that high salt intake can lead to elevation of plasma ADMA. ADMA has been found to be involved in the development of high blood pressure (BP) in Dahl salt-sensitive (DS) hypertensive rats but not in spontaneous hypertensive rats, suggesting that salt might modulate the level of ADMA in salt-sensitive (SS) hypertension independent of blood pressure reduction [13]. Fujiwara *et al.* reported that plasma level of ADMA significantly increased after high salt intake and decreased after salt restriction [14]. Our research team has previously demonstrated that a high salt diet significantly raised plasma ADMA and BP levels while decreasing plasma NO synthesis and urinary NO excretion in normotensive salt-sensitive Chinese people. We deduced that plasma ADMA may be influenced by salt or the alteration of BP [15]. All the facts suggest that salt independent of blood pressure may contribute to ADMA metabolic pathway dysfunction, as well.

However, there is little information on the effects of a high salt diet on the ADMA system independent of blood pressure reduction. The aims of this study were to examine such effects of a high salt diet on the PRMT-1/ADMA/DDAH pathway in Dahl salt-sensitive rats and SS-13^BN^ rats, as well as to explore the mechanisms that regulate ADMA metabolism independent of blood pressure reduction.

## 2. Results

### 2.1. Effects of High Salt Diet on Blood Pressure and Weight of Rats

All rats had normal physiological activities and were healthy during the dietary intervention. At baseline, no significant differences in blood pressure and body weight were detected among groups. The blood pressure levels of rats fed a high salt diet were significantly higher than those of rats from the other groups of the same species. The weights of the SH rats (DS rats given a high salt diet) were significantly higher than those of rats from the other two groups (Table 1).

### 2.2. Measurement of Plasma ADMA and NO_x_

**Figure 1:**
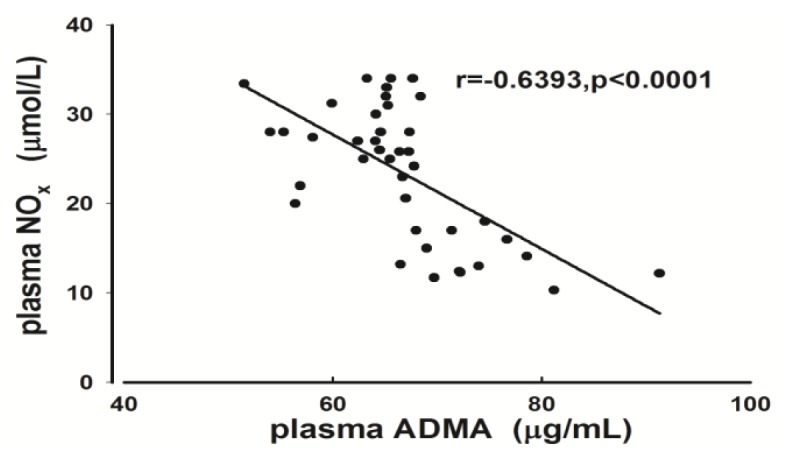
Correlation between plasma ADMA and NO*_x_* in all groups (*r* = −0.6393, *p* < 0.0001).

SH group rats had higher plasma ADMA levels compared with the SN group and the lowest plasma NO*_x_* level among all groups. The plasma levels of NO*_x_* in the SH + HYD (DS rats given a high salt diet and subjected to intragastric administration of hydralazine) group decreased significantly compared to those in the SN group (Table 2). The plasma levels of ADMA were negatively correlated with those of NO*_x_* in all groups (Figure 1).

### 2.3. Activity of DDAH

**Figure 2:**
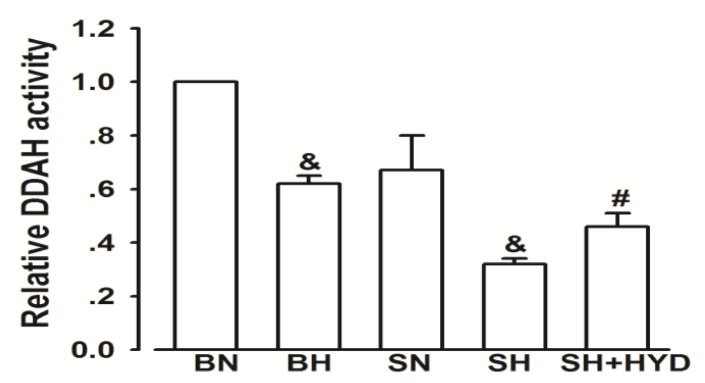
DDAH activity in all groups. (results were normalized to the BN group). ^&^*p* < 0.05 compared with the normal controls. ^#^*p* < 0.05 compared with the SN group and SH group.

DDAH activity of aorta tissues significantly decreased in the high salt group compared with the BN and SN groups. It was significantly lower than in the SN group and higher than in the SH group, respectively (Figure 2).

### 2.4. mRNA Expressions of PRMT-1, DDAH-1, DDAH-2 and eNOS

**Figure 3:**
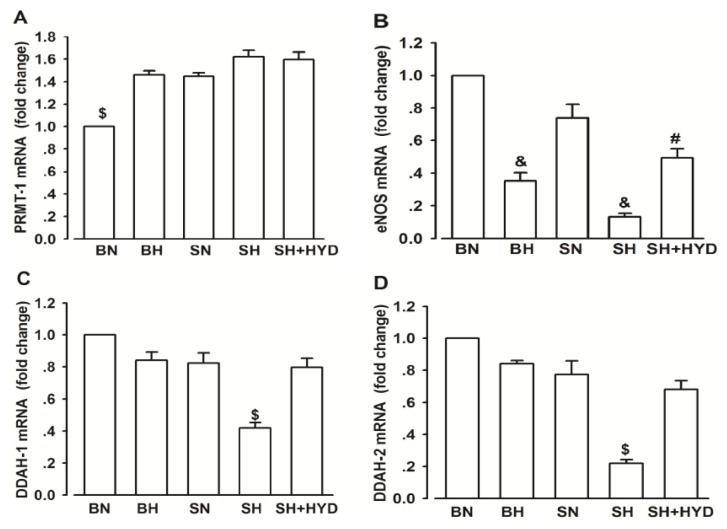
PRMT-1, eNOS and DDAH-1, DDAH-2 mRNA expressions in all groups. (**A**) Relative PRMT-1 mRNA. ^$^*p* < 0.05 compared with the other four groups; (**B**) Relative eNOS mRNA. ^&^*p* < 0.05 compared with the value of normal controls. ^#^*p* < 0.05 compared with the value of SN group and SH group; (**C**) Relative DDAH-1 mRNA. ^$^*p* < 0.05 compared with the other four groups; (**D**) Relative DDAH-2 mRNA. ^$^*p* < 0.05 compared with the other four groups.

Compared with that in the BN group, the mRNA expression of PRMT-1 evidently increased in the four groups, although no significant difference was found among the groups (Figure 3A). The expression of eNOS significantly decreased in BH and SH rats compared with the normal control group, and the quantitative value of the SH + HYD group was significantly lower than the SN group and higher than in SH rats, respectively (Figure 3B). In the SH group, both the expression of DDAH-1 and DDAH-2 were lowest among all the groups (Figure 3C,D).

### 2.5. Protein Expressions of PRMT-1 and eNOS

**Figure 4:**
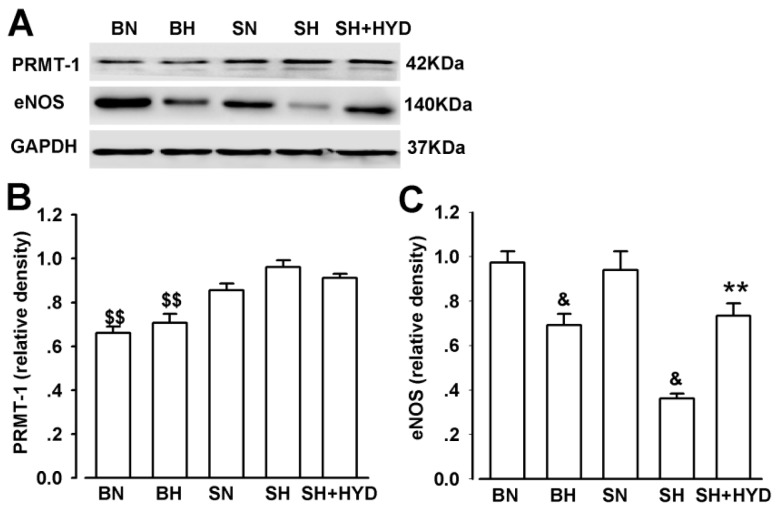
PRMT-1, eNOS protein expressions in all groups. (**A**) Western blot analysis for PRMT-1, eNOS, and GAPDH protein levels in each group tested; (**B**) Relative quantitative analysis of PRMT-1 protein level. ^$$^*p* < 0.05 compared with DS rats; (**C**) Relative quantitative analysis of eNOS protein level. ^&^*p* < 0.05 compared with the normal controls. ** *p* < 0.05, compared with the SN group.

Compared with that in the SS-13^BN^ rat group, the expression of PRMT-1 was significantly raised in DS rats; however, no significant difference among the DS rats was observed (Figure 4B). The expression of eNOS significantly reduced in the BH and SH rats compared with the other three groups, and that of the SH + HYD group was significantly lower compared with the SN group (Figure 4C).

## 3. Discussion

Increasing evidence from multiple clinical studies shows that excess salt intake is related to cardiovascular organ damage, independent of blood pressure [16,17]. Animal studies also demonstrated that administration of a high salt diet contributed to alterations of the structure and function of arteries independent of BP [18,19]. A high salt diet *per se* has major effects on the decreased production of NO on reactivity of arterioles, resistance vessels, aortas of rats and superoxide production [20]. Arterial pressure-independent profibrotic effects of salt in the heart and kidneys have been shown in SHR and WKY rats [21]. In this study, we found salt independent of blood pressure led to ADMA metabolic pathway dysfunction, via a mechanism involving a reduction in the activity of DDAH and expression of eNOS in DS rats.

Our study showed that the blood pressure and body weights of the rats from the SH group were highest among groups. The findings were consistent with a previous study illustrating that Dahl salt-sensitive rats (DS) have a defect in renal pressure, natriuresis, which is associated with retention of sodium and fluid on a high NaCl intake, thereby contributing to hypertension and obesity [22].

NO, which is generated by NO synthase (NOS), plays a pivotal role in regulation of blood pressure and maintenance of vascular function. ADMA can contribute to endothelial dysfunction by inhibition and uncoupling of NOS [23,24]. Our study found that rats of the SH group had the highest plasma ADMA and the lowest plasma NO level among groups, with results indicating that slight ADMA elevation can inhibit NO production significantly. Moreover, plasma levels of NO were lower in the SH+HYD group compared with the SN group. Compared with rats with a normal diet, high salt intake can decrease NO production independent of blood pressure. By contrast, no changes were observed in the BH group. This phenomenon is consistent with the results in previous studies, which demonstrated salt-sensitive Dahl rats possess a functional defect in the production of NO [13,25].

Plenty of evidence has shown that elevated oxidant stress level in the renal medulla and vascular oxidative stress in DS rats contributes to salt-sensitive hypertension [26–28]. PRMT-1 have been shown to be regulated in a redox-sensitive fashion [26]. Moreover, according to a recent study, reactive oxygen species and ADMA form a tightly coupled amplification system, thus aggravating the pathological progression [29]. In our study, the protein expression of PRMT-1 increased significantly in DS rats compared with the 13-BN rats. The expression of PRMT-1 in the SH + HYD group was similar to that in the SN group. This indicated that salt had little impact on PRMT-1 expression independent of blood pressure, probably due to oxidative stress [30,31]. DDAH protects endothelial function by a combination of metabolizing endothelial ADMA and enhancing the expression of eNOS [32,33]. The existing evidence is in accordance with our current observations that DDAH and eNOS exhibited the similar changes in DS rats and SS-13^BN^ rats. In addition, activity of DDAH and expression of eNOS in SH + HYD groups were lower than in the SN group; the relative quantity of the two subtypes presented in a similar trend. The expression of DDAH-2 in the SH group declined to 22.4% compared with the normal control, more than the above value of DDAH-1 (to 42.7%). These results indicated that salt independent of blood pressure significantly affect DDAH (particularly DDAH-2) and eNOS expression, which represented the physiopathological condition of PRMT/ADMA/DDAH pathway dysregulation prior to blood pressure elevation. This could account for our previous finding that endothelial dysfunction already existed in normotensive salt-sensitive subjects [34].

The sole limitation of the study is that the detection result of ADMA concentration was higher than the previous literature had reported. There may be some fault in our experimental operation. However, the main purpose of this study is to compare the impact of high salt intervention on the ADMA system between DS and DR rats, thus the conclusion can only be slightly affected.

We thus propose the following underlying mechanisms: high salt intake leads to increased vascular superoxide production, which may result in impaired DDAH activity and expression. Intracellular ADMA that escapes metabolism reduces endogenous NO production by limiting the availability of eNOS via the uncoupling of eNOS, thereby contributing to oxidative stress in a positive feedback fashion.

## 4. Materials and Methods

### 4.1. Animals and Experimental Procedures

Eight-week-old male Dahl salt-sensitive rats and SS-13^BN^ rats (Charles River Laboratories International, Inc., Wilmington, MA, USA) were bred in a specific pathogen-free animal house. Each group consisted of eight rats. The DS rats were randomly divided into three groups according to their respective diets: normal diet (0.3% NaCl, SN group), high salt diet (8% NaCl, SH group), high salt diet (8% NaCl) and hydralazine (10 mg/kg/d) intragastric administration (SH + HYD group). Similarly, SS-13^BN^ rats were randomly divided into two groups according to their diet: normal diet (0.3% NaCl, BN group), and high salt diet (8% NaCl, BH group). At the end of three weeks, the animals were sacrificed with intraperitoneal injection of 10% chloral hydrate (3 mL/kg); plasma was collected from the abdominal aorta, and the thoracic aorta were immediately snap-frozen in liquid nitrogen and kept frozen at −80 °C until biochemical determinations and *in vitro* studies. The experiments were approved by the Institutional Animal Ethics Committee of Xi’an Jiaotong University.

### 4.2. Measurement of Blood Pressure and Body Weight

Before the intervention period and last day of each week, the BP of conscious rats were measured using the tail–cuff plethysmography with a computerized system (Amplifier Model 229, IITC Life Science, Woodlands, CA, USA). All rats did treadmill exercise and were adjusted to the blood pressure measurements by tail–cuff plethysmography prior to the final measurements. A 13 mm, the cuff was placed around the tail of rats and inflated to 240 mm Hg. Systolic pressure was recorded as the pressure at the point when the first tail pulse was detected. Systolic blood pressure was measured four times, every 3 min, and the average value was calculated. Body weights were measured at the end of three weeks.

### 4.3. Measurement of Plasma ADMA

The concentration of ADMA was determined by high-performance liquid chromatography (HPLC). Reagents and ADMA were purchased from Sigma (St. Louis, MO, USA). The serum samples (200 μL) of rats mixed with chromatographic grade acetonitrile (400 μL). The supernatant was collected after centrifugation at 12,000 rpm for 10 min at 4 °C. The supernatant (100 μL) was diluted with 0.1 mol/L hydrochloric acid (HCL) to 1.5 mL. The samples (20 μL) and standards (20 μL) were incubated for 3 min with *o*-phthaldialdehyde reagent (10 mg/mL OPA in borate buffer, pH 9.0, containing 0.4% mercaptoethanol). A Shimaduz LC-10A liquid chromatograph equipped with a Model 7125i injection valve and a Shimaduz RF-10A_XL_ fluorescence detector was used. A 5 μM Waters Symmetry C18 (5 μm; 150 × 3.9 mm) coupled to a Waters Sentry Symmetry C18 guard column (5 μm; 3.9 mm × 20 mm) was operated at room temperature. The mobile phase was 77:23 (*v*/*v*) potassium–phosphate buffer (pH 3.5): acetonitrile. The flow rate was 1.0 mL/min. The injection volume was 20 μL. The derivative fluorescence intensity was detected at the excitation and emission wavelengths of 338 and 447 nm, respectively. Data acquisition and analysis were performed on data processor N2010 (Zhejiang University, Hangzhou, China). Within-assay and between-assay coefficients of variation for ADMA were 1.8% and 2.5%, respectively, and the detection limit of the assay was 0.15 μmol/L.

### 4.4. Measurement of Plasma Nitrite/Nitrate (NO_x_)

The plasma NO*_x_* production of rats was measured by the Griess method according to the indication on the NO*_x_* assay kit (Beyotime Institute of Biotechnology, Haimen, China). After the serum of each rat was incubated according to the aforementioned grouping, the supernate (50 μL) was mixed with an equal volume (50 μL) of Griess reagents I and II at room temperature for 15 min. NO*_x_* concentration was determined by spectrophotometry (560 nm, Biotech Engineering Ltd., Falmouth, UK) from a standard curve (0–100 mmol/L) derived from NaNO_2_.

### 4.5. Measurement of DDAH Activity

According to the theory that DDAH can catalyze ADMA to dimethylamine and l-citrulline [35], the activity of DDAH was assayed by content of l-citrulline generated in the reaction. The tissue homogenates of each group were incubated with 1 mM ADMA and 0.1 M sodium–phosphate buffer (pH 6.5) in a total volume of 0.5 mL for 30 min at 37 °C. The supernatant was incubated with diacetyl monoxime (0.8%) and antipyrine (0.5%) at 60 °C for 100 min. The amounts of l-citrulline formed were determined by spectrophotometric analysis at 460 nm. One unit of the enzyme was defined as the amount that catalyzed the formation of 1 mmol/L l-citrulline from ADMA per minute at 37 °C.

### 4.6. RNA Quantitation

Total RNA was isolated by Trizol extraction (Invitrogen, Carlsbad, CA, USA). cDNA was synthesized with RevertAid™ First Strand cDNA Synthesis Kit (Fermentas, Burlington, Ontario, Canada). The mRNA expressions of PRMT-1, DDAH-1, DDAH-2 and endothelial NOS (eNOS) were tested by real-time quantitative PCR, which was performed on an iQ5 real-time PCR detection system (Bio-Rad Laboratories, Hercules, CA, USA) with SYBR PremixEx Taq™ II (Takara, Shiga, Japan). The thermo cycling conditions were 3 min at 95 °C followed by 40 cycles of 10 s at 95 °C, 30 s at 58 °C and 30 s at 72 °C. The data were also collected at the annealing step (72 °C) of each cycle. The relative gene expression was normalized by GAPDH. Data were analyzed on the basis of the relative expression method with the formula relative expression 2^−ΔΔCT^, where ΔΔCT is ΔCt (different group) − ΔCt (control group), ΔCt is Ct (target gene) − Ct (GAPDH), and Ct is the cycle at which the threshold is crossed. The results were normalized to the BN group. The information of primer (Sangon, Shanghai, China) is shown in Table 3.

### 4.7. Western Blot Analysis

The aorta tissues were homogenized and lysed with RIPA lysis buffer (Genshare, Shannxi, China). The supernatant was collected after centrifugation at 14,000 rpm for 15 min at 4 °C. Proteins were quantified by the Bradford assay with a protein assay kit. Equal amounts of protein samples (45 μg) were separated in 12% SDS-PAGE gels, and transferred to nitrocellulose membranes at 250 mA for 2 h. Non-specific binding was blocked with 5% skim milk for 2 h at room temperature. Immunoblotting was performed for PRMT-1 (1:1000; Abcam, Cambridge, UK), eNOS (1:1000; CST, Beverly, MA, USA), and GAPDH (1:5000; Bioworld, Louis Park, USA). Horseradish peroxidase-conjugated anti-rabbit (1:5000, Thermo Scientific, MA, USA), anti-rat (1:5000, Thermo Scientific, MA, USA) antibodies were used. The membrane was finally washed with TBST. Densitometric analysis was performed with Bio Rad-IQ5 Image Software, and the relative ratio to the GAPDH expression was calculated for each sample.

### 4.8. Statistical Analysis

Data were presented as mean ± S.E.M. Differences between treatment groups were compared by unpaired *t* test or one-way analysis of variance (ANOVA), followed by the Student–Newman–Keuls *post hoc* test for multiple comparisons. A probability (*p*) value of < 0.05 was considered significant.

## 5. Conclusion

In conclusion, a high salt diet independent of blood pressure level decreases plasma NO by downregulation of DDAH activity and eNOS expression in aorta of DS rats; our findings indicated that prior to blood pressure elevation, there must already exist dysregulation of the DDAH/ADMA/eNOS system in normotensive salt-sensitive subjects. The study sheds new light on understanding the pathogenesis of salt-sensitive hypertension. The enhancement of DDAH activity or upregulation of DDAH expression may become a novel therapeutic strategy for early prevention of cardiovascular diseases.

## Figures and Tables

**Table 1:** Blood pressure and weight of the rats after dietary intervention.

Group	SBP (mmHg)	Weight (g)
13-BN normal diet (*n* = 8)	138.6 ± 4.8	345.3 ± 19.3
13-BN high salt diet (*n* = 8)	147.6 ± 5.1 *	342.6 ± 5.1
SS normal diet (*n* = 8)	147.3 ± 12.6	321.8 ± 17.1
SS high salt diet (*n* = 8)	160.2 ± 10.3 **	365.5 ± 19.4 **
SS high salt diet + hydralazine (*n* = 8)	144.7 ± 5.9	316.7 ± 17.7

* *p* < 0.05, compared with the BN(SS-13^BN^ rats given a normal salt diet) group.

** *p* < 0.05, compared with the SN (DS rats given a normal salt diet) group.

**Table 2:** Plasma ADMA and NO*_x_* levels of the rats.

Group	Plasma ADMA (μg/mL)/(μmol/L)	Plasma NO*_x_* (μmol/L)
13-BN normal diet (*n* = 8)	64.7 ± 4.3 (320.3 ± 21.3)	25.6 ± 2.3
13-BN high salt diet (*n* = 8)	67.5 ± 8.8 (334.2 ± 43.6)	20.2 ± 3.2
SS normal diet (*n* = 8)	61.4 ± 5.3 (304.0 ± 26.2)	30.1 ± 2.4
SS high salt diet (*n* = 8)	75.7 ± 12.1 (374.8 ± 74.6) **	12.4 ± 1.7 $
SS high salt diet + hydralazine (*n* = 8)	65.2 ± 2.4 (322.8 ± 11.9)	20.3 ± 4.6 **

** *p* < 0.05, compared with the SN group.

$ *p* < 0.05, compared with the other four groups.

**Table 3:** Information of primers for real-time quantitative PCR.

Gene	Nucleotide sequence	
GAPDH	Forward primer	ATGGTGAAGGTCGGTGTGAACG
	Reverse primer	CGCTCCTGGAAGATGGTGATGG

PRMT-1	Forward primer	GTGACAGCCATTGAGGACCG
	Reverse primer	TGTGGCATCGGGTGAACTCG

DDAH-1	Forward primer	ACAGTCCCCGTGGCCGATTCTT
	Reverse primer	TGGGGTTCGGTGCAGCAAGA

DDAH-2	Forward primer	GACACGGCTCTAATCACAAG
	Reverse primer	AGCGTAGCGTTCTCATCC

eNOS	Forward primer	TTGGACAAGTCCTCACCGCC
	Reverse primer	TGGGTGCGCAATGTGAGTCC
